# Supplementary material for: Approximate Cross-Validated Mean Estimates for Bayesian Hierarchical Regression Models
Source: J Comput Graph Stat. Author manuscript; Available in PMC 2025 Aug 6. (PMC12327438; doi:10.1080/10618600.2024.2404711)
Supplement: Supp 1 [file NIHMS2025082-supplement-Supp_1.zip › AXE_supplementary/AXE_supplementary/AXE_appendix.pdf]

# Appendix for "Approximate cross-validated mean estimates for Bayesian hierarchical regression models"


Amy Zhang
Department of Statistics, Pennsylvania State University
and
Michael J. Daniels
Department of Statistics, University of Florida
and
Changcheng Li
School of Mathematical Sciences, Dalian University of Technology
and
Le Bao
Department of Statistics, Pennsylvania State University


July 23, 2024

## A Proof for Theorem 2.1

For reference, we repeat here relevant notation used throughout the paper. $Y \in \mathbb{R}^N$ denotes a continuous response vector and $X \in \mathbb{R}^{N \times P}$ denotes the design matrix. We refer to the regression coefficients as $\beta := (\beta_1', \beta_2')' \in \mathbb{R}^P$, which includes fixed effects $\beta_1$ and random effects $\beta_2$. $\Sigma \in \mathbb{R}^{P_2 \times P_2}$ is the covariance matrix for the random effects $\beta_2$. $\phi \in \mathbb{R}^+$ is a positive scalar denoting the standard deviation of the residuals. $\hat{\phi}$, $\hat{\Sigma}$, and $\hat{\beta}$ denote the full-data posterior mean estimates $E[\phi|Y]$, $E[\Sigma|Y]$, and $E[\beta|Y]$, respectively. For $J$-fold cross-validation, $j = 1, \ldots, J$ indicates the cross-validation folds; $\theta_j \in \beta_2$, $\theta_j \in \mathbb{R}^{P_j}$, $P_j \geq 1$, refers to the test data-specific random effects such; $s_j$ corresponds to the indices

of the test data for CV fold $j$; $Y_{s_j} \in I\!R^{n_j}$ is the test response data; $Y_{-s_j} \in I\!R^{N-n_j}$ the training response data; and similarly $X_{s_j} \in I\!R^{n_j \times P}$ refers to the rows of $X$ indexed by $s_j$, while $X_{-s_j} \in I\!R^{(N-n_j)\times P}$ refers to the remaining rows of $X$ without $X_{s_j}$. $\beta_{/\theta_j}$ refers to those coefficients without the test data-specific random effects, inclusive of $\theta_{-j}$. We denote the transpose of a matrix (or vector) $A$ as $A'$.

In addition, in the proof we refer to $X_{-s_j}E[\beta|\Sigma, \phi, Y]$, the conditional expected value based on the full data $Y$, as $(Z)_{-s_j}$. We denote the mean value across all entries $Y_{s_j}$ as $\bar{Y}_{s_j} := n_j^{-1}\sum_{i\in s_j}^{N} Y_i$. The mean residual for cluster $Y_{s_J}$ is denoted as $\bar{e}_{s_j} = n_j^{-1}\sum_{i\in s_j}(Z_i - Y_i)$, where $x_i'$ is the $i^{th}$ row of $X$.

We denote the difference in log-likelihoods $\ell(\Sigma, \phi|Y_{-s_j}) - \ell(\Sigma, \phi|Y)$ as $\Delta_j$, where $\ell(\Sigma, \phi|Y_{-s_j})$ and $\ell(\sigma, \phi|Y)$ are the log posterior densities of $\Sigma$ and $\phi$ given $Y_{-s_j}$ and $Y$ respectively. And let $B := [\mathbf{0}\ \ I_{P_2}] \in I\!R^{P_2\times P}$, where $\mathbf{0} \in I\!R^{P_2\times(P-P_2)}$ is the matrix of 0s and $I_{P_2} \in I\!R^{P_2\times P_2}$ is the $P_2$-dimensional identity matrix and $\mathbf{0}$ the $\mathbf{0}$ is the $P_2 \times (P-P_2)$-dimensional matrix of 0s such that $B$ has $P$ columns.

The new terms listed above are also re-defined when introduced in the proof.

**Lemma A.1.** *Let response vector $Y \in I\!R^N$ of a hierarchical linear regression follow a normal distribution as in (1) and let $s_j$ be the set of indices of $\theta_j$ in $\theta$ such that $X_{s_j}$ is made up of identical rows. Let $x_{s_j}'$ refer to a row in $X_{s_j}$. $V$ is defined as in (3). We list several facts pertaining to $V$ and $V_{-s_j}$.*

1. $V_{-s_j} = V + \frac{n_j}{\phi^2}\frac{1}{1-\frac{n_j}{\phi^2}x_{s_j}'Vx_{s_j}}Vx_{s_j}x_{s_j}'V$

2. $V \preceq V_{-s_j}$, *where $V \preceq V_{-s_j}$ indicates that $V_{-s_j} - V$ is positive semi-definite*

3. $\frac{n_j}{\phi^2}x_{s_j}'Vx_{s_j} \leq 1$

4. $x_{s_j}'V_{-s_j}x_{s_j} = x_{s_j}'Vx_{s_j}\frac{1}{1-\frac{n_j}{\phi^2}x_{s_j}'Vx_{s_j}} \geq x_{s_j}'Vx_{s_j}$

5. $\det\left(V_{-s_j}\right) = \det(V)\frac{1}{1-\frac{n_j}{\phi^2}x_{s_j}'Vx_{s_j}} \geq \det(V)$

6. $\frac{1}{\phi^4}Y_{-s_j}'X_{-s_j}V_{-s_j}X_{-s_j}'Y_{-s_j} = \frac{1}{\phi^4}Y'XVX'Y - \frac{n_j}{\phi^2}\bar{Y}_{s_j}^2 + \frac{\bar{e}_{s_j}^2}{\phi^2/n_j}\frac{1}{1-\frac{n_j}{\phi^2}x_{s_j}'Vx_{s_j}}$, *where $\bar{Y}_{s_j}$ denotes the mean value across all entries $Y_{s_j}$, $\bar{Y}_{s_j} = n_j^{-1}\sum_{i\in s_j}^{N} Y_i$ and $\bar{e}_{s_j} = n_j^{-1}\sum_{i\in s_j}(Z_i - Y_i)$ denotes the mean residual for cluster $Y_{s_j}$.*

*Remark* 1. Note that our theoretical results (Theorem 2.1 and Corollary 2.2) are both treating the $j$-th CV fold as a whole. From the theoretical setting in Theorem 2.1, the $j$-th CV fold contains all the observations informing $\theta_j$. Hence as $J$ increases, more CV folds are added sequentially. Suppose $V$ is the $V$ matrix as defined in (3) in the main text when the number of CV folds is $J$, we can see that $V_{-s_J}$ is actually the $V$ matrix when the number of CV folds is $J-1$. From item 2 in Lemma A.1, we know that the $V_J \preceq V_{J-1}$, where $V_{J-1}$ and $V_J$ are the $V$ matrices when the numbers of CV folds are $J-1$ and $J$, respectively, and $A \preceq B$ for matrices $A$ and $B$ indicates that $B - A$ is positive semidefinite. And by mathematical induction we can see that $V_J \preceq V_{J-1} \preceq \cdots \preceq V_2 \preceq V_1$. Hence the $V$ matrix is non-increasing as $J$ increases in the sense of $\preceq$. Similarly, from item 5 we can also see that $\det(V)$ is also non-increasing, and from item 2 and 4 $x'_{s_j} V x_{s_j}$ is non-increasing as $J$ goes to infinity.

*Proof.* 1. This follows from using the Sherman-Morrison formula on $V_{-s_j} = (V^{-1} - \frac{n_j}{\phi^2} x_{s_j} x'_{s_j})^{-1}$. The Sherman-Morrison formula states that given an invertible square matrix $A \in I\!R^{P\times P}$ and vectors $u, v \in I\!R^P$, if $A + uv'$ is invertible, then

$$(A + uv')^{-1} = A^{-1} - \frac{A^{-1}uv'A^{-1}}{1 + v'A^{-1}u}.$$

The result follows when $A = V^{-1}$, $u = -\frac{n_j}{\phi^2} x_{s_j}$, $v = x_{s_j}$.

2. Using a similar process as in 1., if we re-write $V$ in terms of $V_{-s_j}$, we obtain $V = V_{-s_j} - \frac{n_j}{\phi^2} \frac{1}{1+\frac{n_j}{\phi^2} x'_{s_j} V_{-s_j} x_{s_j}} V_{-s_j} x_{s_j} x'_{s_j} V_{-s_j}$. The difference, $\frac{n_j}{\phi^2} \frac{1}{1+\frac{n_j}{\phi^2} x'_{s_j} V_{-s_j} x_{s_j}} V_{-s_j} x_{s_j} x'_{s_j} V_{-s_j}$, is positive semi-definite and so $V_{-s_j} \succeq V$, where $A \succeq B$ is defined as $A - B$ is positive semi-definite.

3. From 2., we note that $V_{-s_j} \succeq V$ and from 1., $V_{-s_j} - V$ is $\frac{n_j}{\phi^2} \frac{1}{1-\frac{n_j}{\phi^2} x'_{s_j} V x_{s_j}} V x_{s_j} x'_{s_j} V$. The fact that $V$ is positive-definite implies $\frac{n_j}{\phi^2} x'_{s_j} V x_{s_j} > 0$, then for $V_{-s_j} - V$ to be positive semi-definite, $\frac{n_j}{\phi^2} x'_{s_j} V x_{s_j} \leq 1$ must be true.

4. Using 1., $x'_{s_j} V_{-s_j} x_{s_j} = x'_{s_j} V x_{s_j} + \frac{n_j}{\phi^2} \frac{1}{1-\frac{n_j}{\phi^2} x'_{s_j} V x_{s_j}} (x'_{s_j} V x_{s_j})^2 = x'_{s_j} V x_{s_j} (1 - \frac{n_j}{\phi^2} x'_{s_j} V x_{s_j})^{-1}$. The inequality follows from 3. , as $1 - \frac{n_j}{\phi^2} x'_{s_j} V x_{s_j} \leq 1$.

5. As the held-out data correspond to identical rows of $X$, we have a closed-form

solution for the determinant of $V_{-s_j}$ in terms of $V$:

$$\begin{aligned}
\det(V^{-1} - \phi^{-2}X'_{s_j}X_{s_j}) &= \det(V^{-1})\det(1 - \frac{n_j}{\phi^2}x'_{s_j}Vx_{s_j}) && \textit{Sylvester's det. theorem} \\
&= \det(V^{-1})(1 - \frac{n_j}{\phi^2}x'_{s_j}Vx_{s_j}) \\
\implies \det(V_{-s_j}) &= \det(V)\frac{1}{1 - \frac{n_j}{\phi^2}x'_{s_j}Vx_{s_j}} \\
&\geq \det(V) && \textit{from } 3.
\end{aligned}$$

6. We first show that $\frac{1}{\phi^2}V_{-s_j}X'_{-s_j}Y_{-s_j} = \frac{1}{\phi^2}VX'Y + \frac{n_j}{\phi^2}\frac{\bar{e}_{s_j}}{1-\frac{n_j}{\phi^2}x'_{s_j}Vx_{s_j}}Vx_{s_j}$. Let $(Z)_{-s_j}$ refer to $X_{-s_j}E[\beta|\Sigma,\phi,Y]$, the conditional expected value based on the full data $Y$:

$$\begin{aligned}
\frac{1}{\phi^2}V_{-s_j}X'_{-s_j}Y_{-s_j} &= \frac{1}{\phi^2}[V + \frac{n_j}{\phi^2}\frac{1}{1 - \frac{n_j}{\phi^2}x'_{s_j}Vx_{s_j}}Vx_{s_j}x'_{s_j}V](X'Y - X'_{s_j}Y_{s_j}) && \textit{from } 1. \\
&= \frac{1}{\phi^2}VX'Y + Vx_{s_j}\left[-\frac{n_j}{\phi^2}\bar{Y}_{s_j} + \frac{n_j}{\phi^2}\frac{1}{1 - \frac{n_j}{\phi^2}x'_{s_j}Vx_{s_j}}\left(Z_{s_j} - \frac{n_j}{\phi^2}x'_{s_j}Vx_{s_j}\bar{Y}_{s_j}\right)\right] \\
&= \frac{1}{\phi^2}VX'Y - Vx_{s_j}\left[\frac{n_j}{\phi^2}\frac{1}{1 - \frac{n_j}{\phi^2}x'_{s_j}Vx_{s_j}}\bar{Y}_{s_j} - \frac{n_j}{\phi^2}\frac{1}{1 - \frac{n_j}{\phi^2}x'_{s_j}Vx_{s_j}}Z_{s_j}\right] \\
&= \frac{1}{\phi^2}VX'Y + \frac{n_j}{\phi^2}\frac{\bar{e}_{s_j}}{1 - \frac{n_j}{\phi^2}x'_{s_j}Vx_{s_j}}Vx_{s_j}.
\end{aligned}$$

Following a similar procedure, we can re-write $\frac{1}{\phi^4}Y'_{-s_j}X_{-s_j}V_{-s_j}X'_{-s_j}Y_{-s_j}$ in terms of its full-data counterpart, $\frac{1}{\phi^4}Y'XVX'Y$, along with an additional difference term. Let $(Z)_{-s_j}$

refer to $X_{-s_j}E[\beta|\Sigma,\phi,Y]$, the conditional expected value based on the full data $Y$.

$$\begin{aligned}
\frac{1}{\phi^4}Y'_{-s_j}X_{-s_j}V_{-s_j}X'_{-s_j}Y_{-s_j} &= \frac{1}{\phi^4}Y'_{-s_j}X_{-s_j}VX'Y + \frac{n_j}{\phi^4}\frac{\bar{e}_{s_j}}{1-\frac{n_j}{\phi^2}x'_{s_j}Vx_{s_j}}Y'_{-s_j}X_{-s_j}Vx_{s_j} \\
&= \frac{1}{\phi^2}Y'_{-s_j}(Z)_{-s_j} + \frac{n_j}{\phi^2}\frac{\bar{e}_{s_j}}{1-\frac{n_j}{\phi^2}x'_{s_j}Vx_{s_j}}\left(\frac{1}{\phi^2}Y'XVx_{s_j} - \frac{1}{\phi^2}Y'_{s_j}X_{s_j}Vx_{s_j}\right) \\
&= \frac{1}{\phi^2}Y'_{-s_j}(Z)_{-s_j} + \frac{n_j}{\phi^2}\frac{\bar{e}_{s_j}}{1-\frac{n_j}{\phi^2}x'_{s_j}Vx_{s_j}}\left(Z_{s_j} - \frac{n_j}{\phi^2}x'_{s_j}Vx_{s_j}\bar{Y}_{s_j}\right) \\
&= \frac{1}{\phi^2}Y'_{-s_j}(Z)_{-s_j} + \bar{e}_{s_j}\frac{n_j}{\phi^2}\left[\frac{1}{1-\frac{n_j}{\phi^2}x'_{s_j}Vx_{s_j}}\left(Z_{s_j} - \frac{n_j}{\phi^2}x'_{s_j}Vx_{s_j}\bar{Y}_{s_j}\right) + \bar{Y}_{s_j} - \bar{Y}_{s_j}\right] \\
&= \frac{1}{\phi^2}Y'_{-s_j}(Z)_{-s_j} + \bar{e}_{s_j}\frac{n_j}{\phi^2}\left[\bar{Y}_{s_j} + \frac{\bar{e}_{s_j}}{1-\frac{n_j}{\phi^2}x'_{s_j}Vx_{s_j}}\right] \\
&= \frac{1}{\phi^2}Y'_{-s_j}(Z)_{-s_j} + \bar{e}_{s_j}\frac{n_j}{\phi^2}\left[\bar{Y}_{s_j} + \frac{\bar{e}_{s_j}}{1-\frac{n_j}{\phi^2}x'_{s_j}Vx_{s_j}}\right] - \frac{n_j}{\phi^2}\bar{Y}_{s_j}Z_{s_j} + \frac{n_j}{\phi^2}\bar{Y}_{s_j}Z_{s_j} \\
&= \frac{1}{\phi^4}Y'XVX'Y - \frac{n_j}{\phi^2}\bar{Y}^2_{s_j} + \frac{\bar{e}^2_{s_j}}{\phi^2/n_j}\frac{1}{1-\frac{n_j}{\phi^2}x'_{s_j}Vx_{s_j}}.
\end{aligned}$$

□

**Lemma A.2.** *Let response vector $Y \in I\!R^N$ of a hierarchical linear regression follow a normal distribution as in (1) and let $s_j$ be the set of indices of $\theta_j$ in $\theta$ such that $X_{s_j}$ is made up of identical rows. $V$ is defined as in (3). The difference $\Delta_j$ between the log densities $\ell(\Sigma,\phi|Y_{-s_j})$ and $\ell(\Sigma,\phi|Y)$ is*

$$\begin{aligned}
\Delta_j &:= \ell(\Sigma,\phi|Y_{-s_j}) - \ell(\Sigma,\phi|Y) \\
&= C + n_j\log\phi - \frac{1}{2}\log\left(1-\frac{n_j}{\phi^2}x'_{s_j}Vx_{s_j}\right) - \frac{n_j}{\phi^2}\bar{Y}^2_{s_j} + \frac{\bar{e}^2_{s_j}}{\phi^2/n_j}\frac{1}{1-\frac{n_j}{\phi^2}x'_{s_j}Vx_{s_j}},
\end{aligned}$$

*where $C \in I\!R$ is a constant that does not involve $\Sigma$ or $\phi$, and $\ell(\Sigma,\phi|Y_{-s_j})$ and $\ell(\sigma,\phi|Y)$ are the log posterior densities of $\Sigma$ and $\phi$ given $Y_{-s_j}$ and $Y$ respectively.*

*Proof.* The log-likelihood is as follows:

$$\ell(\Sigma,\phi|Y) \propto \log f_\phi(\phi) + \log f_\Sigma(\Sigma) - N\log(\phi) - \frac{1}{2}\log\det(\Sigma) + \frac{1}{2}\log\det(V) + \frac{1}{2\phi^4}Y'XVX'Y.$$

Using Lemma A.1, items 5 and 6, we can directly obtain the difference between the log-likelihoods $\ell(\Sigma,\phi|Y_{-s_j}) - \ell(\Sigma,\phi|Y)$ as stated. □

**Lemma A.3.** *Let response vector $Y \in I\!R^N$ of a hierarchical linear regression follow a normal distribution as in (1) and let $V$ defined as in (3). Let $B := [\mathbf{0} \;\; I_{P_2}] \in I\!R^{P_2 \times P}$, where $\mathbf{0} \in I\!R^{P_2 \times (P-P_2)}$ is a matrix of 0s and $I_{P_2} \in I\!R^{P_2 \times P_2}$ is the $P_2$-dimensional identity matrix such that $B$ has $P$ columns. The partial derivative $\frac{\partial}{\partial \Sigma} a'Vc$ is $\Sigma^{-1}BVac'VB'\Sigma^{-1}$ for vectors $a \in I\!R^P$, $c \in I\!R^P$.*

*Proof.*

$$\begin{aligned} \frac{\partial}{\partial \Sigma_{s_j i}} V &= -V \left( \frac{\partial}{\partial \Sigma_{s_j i}} V^{-1} \right) V \\ &= VB'\Sigma^{-1}\delta_i \delta_j' \Sigma^{-1} BV, \end{aligned}$$

where $\delta_i \in I\!R^{P_2}$ is the binary vector with a 1 only at the $i^{th}$ index. Then,

$$\begin{aligned} \frac{\partial}{\partial \Sigma_{s_j i}} a'Vc &= \frac{\partial}{\partial \Sigma_{s_j i}} \operatorname{tr}(a'Vc) \\ &= \operatorname{tr}\left( ca' \frac{\partial}{\partial \Sigma_{s_j i}} V \right) \\ &= \operatorname{tr}\big( ca'VB'\Sigma^{-1}\delta_i\delta_j'\Sigma^{-1}BV \big) \\ &= \operatorname{tr}\big( \delta_j'\Sigma^{-1}BVca'VB'\Sigma^{-1}\delta_i \big) \\ &= \delta_j'\Sigma^{-1}BVca'VB'\Sigma^{-1}\delta_i, \end{aligned}$$

as the trace is invariant under cyclic permutations.

Then

$$\frac{\partial}{\partial \Sigma} a'Vc = \Sigma^{-1}BVac'VB'\Sigma^{-1}.$$

□

We re-state Theorem 2.1 below:

Let response vector $Y \in I\!R^N$ of a hierarchical linear regression follow a normal distribution as in (1) and (2) and define $\theta$ as in (7). The data are partitioned into $J$ CV folds based on $\theta$, where all data informing $\theta_j$ correspond to the test data for CV fold $j$, and $s_j \subset \{1, \ldots, N\}$ is the set of indices for the test data in the $j^{th}$ CV fold. Let $X_{s_j} = \mathbb{1}_{n_j} x_j'$ for some vector $x_j \in I\!R^P$, where $\mathbb{1}_{n_j}$ is a vector of 1s with length $n_j$, $n_j$ is the size of $s_j$,

and $n_j \geq 1$, $\mathbb{1} \in \text{span}(X)$, and $f_\Sigma(\Sigma)$ and $f_\phi(\phi)$ are proper prior densities. $V$ is defined as in (3). As $J$ goes to infinity (and thus $N$ goes to infinity), we have

$$\begin{aligned} |E[\phi|Y_{-s_j}]^{-1}E[\phi|Y] - 1| &= \mathcal{O}_P\left(n_j/N\right), \\ \|E[\Sigma|Y_{-s_j}]^{-1}E[\Sigma|Y] - I\| &= \mathcal{O}_P\left(n_j/N\right), \end{aligned}$$

where $I$ is the identity matrix, $\|.\|$ is the operator norm of a matrix defined as $\|A\| = \sqrt{\lambda_1(A'A)}$ and $\lambda_1$ is the largest eigenvalue.

*Proof.* The log-likelihood is as follows:

$$\ell(\Sigma, \phi|Y) \propto \log f_\phi(\phi) + \log f_\Sigma(\Sigma) - N\log(\phi) - \frac{1}{2}\log\det(\Sigma) + \frac{1}{2}\log\det(V) + \frac{1}{2\phi^4}Y'XVX'Y.$$

We note that, other than the prior densities whose forms are unknown, the log-likelihood consists of terms with $P_2$ or $N$ summands. And $N^{-1}\ell(\Sigma, \phi|Y)$ is finite, and we can say that for any $N$, the solution to $N^{-1}\frac{\partial}{\partial\Sigma}\ell(\Sigma, \phi|Y) = 0$ is the same as the solution to $\frac{\partial}{\partial\Sigma}\ell(\Sigma, \phi|Y) = 0$:

$$\operatorname*{argmax}_{\Sigma,\phi} \ell(\Sigma, \phi|Y) = \operatorname*{argmax}_{\Sigma,\phi} \frac{\ell(\Sigma, \phi|Y)}{N}.$$

Note that as $J$ goes to infinity, the dimensions of $\Sigma$, $V$, and $Y$ likewise increase. Let $a_{s_j}$ be the sequence of operator norms of differences between $\frac{\partial}{\partial\Sigma}\frac{\ell(\Sigma,\phi|Y)}{N}$ and $\frac{\partial}{\partial\Sigma}\frac{\ell(\Sigma,\phi|Y_{-s_j})}{N}$:

$$a_{s_j} = \left\| \frac{\partial}{\partial\Sigma}\frac{\ell(\phi, \Sigma|Y_{-s_j})}{N} - \frac{\partial}{\partial\Sigma}\frac{\ell(\phi, \Sigma|Y)}{N} \right\|.$$

If we show $a_{s_j} \to 0$ as $J \to \infty$, then we also show that $|\operatorname{argmax}_\Sigma \ell(\Sigma, \phi|Y_{-s_j}) - \operatorname{argmax}_\Sigma \ell(\Sigma, \phi|Y)| \to 0$ as $J \to \infty$.

Let $\Delta_j$ be the difference in log-likelihoods stated in Lemma A.2:

$$\begin{aligned} a_{s_j} &= \left\| \frac{\partial}{\partial\Sigma}\frac{\ell(\phi, \Sigma|Y_{-s_j})}{N} - \frac{\partial}{\partial\Sigma}\frac{\ell(\phi, \Sigma|Y)}{N} \right\| \\ &= \left\| \frac{\partial}{\partial\Sigma}\left(\frac{\ell(\phi, \Sigma|Y)}{N} - \frac{\Delta_j}{N}\right) - \frac{\partial}{\partial\Sigma}\frac{\ell(\phi, \Sigma|Y)}{N} \right\| \\ &= \left\| \frac{1}{N}\frac{\partial}{\partial\Sigma}\Delta_j \right\|. \end{aligned}$$

It remains to show that $\left\|\frac{\partial}{\partial\Sigma}\Delta_j\right\|/N = \mathcal{O}\left(n_j/N\right)$. Of the terms in $\Delta_j$, only those involving $V$ are dependent on $J$ and $N$. The derivative is as follows:

$$\frac{\partial}{\partial\Sigma}\Delta_j = \frac{n_j/\phi^2}{1 - \frac{n_j}{\phi^2}x'_{s_j}Vx_{s_j}}\left(\frac{1}{2}\left(1 + \frac{\bar{e}^2_{s_j}}{1 - \frac{n_j}{\phi^2}x'_{s_j}Vx_{s_j}}\right)\frac{\partial}{\partial\Sigma}x'_{s_j}Vx_{s_j} + \bar{e}_{s_j}\frac{\partial}{\partial\Sigma}x'_{s_j}VX'Y\right). \quad (1)$$

Note that $\left\|\frac{\partial}{\partial\Sigma}\Delta_j\right\|/N = \mathcal{O}\left(n_j/N\right)$ is trivially true when $x_{s_j} = 0$, so without loss of generality, we assume that $x_{s_j} \neq 0$ for all $j$.

Furthermore, examining each of the terms in $\frac{\partial}{\partial\Sigma}\Delta_j$ as $N$ and $J$ increase, we know from Lemma A.1, items 2 and 4, that $V$ and $\frac{1}{1-\frac{n_j}{\phi^2}x'_{s_j}Vx_{s_j}}$ are non-increasing as $N$ and $J$ increase, with $\frac{1}{1-\frac{n_j}{\phi^2}x'_{s_j}Vx_{s_j}} > 0$. Also see Remark 1 of Lemma A.1. We also note that $\sqrt{n_j}\bar{e}_{s_j} = \mathcal{O}_P\left(\phi\right)$.

By Lemma A.3, $\frac{\partial}{\partial\Sigma}x'_{s_j}Vx_{s_j} = \Sigma^{-1}BVx_{s_j}x'_{s_j}VB'\Sigma^{-1}$, where $a = c = x_{s_j}$. Again we know from Lemma A.1, item 2 that this value does not increase with $N$ and $J$. Likewise by Lemma A.3, $\frac{\partial}{\partial\Sigma}x'_{s_j}VX'Y = \Sigma^{-1}BVx_{s_j}Y'XVB'\Sigma^{-1}$, where $a = x_{s_j}$ and $c = X'Y$. Note that $Y'XVB' = E[\beta|\Sigma,\phi,Y]$, which is $\mathcal{O}_P\left(1\right)$ elementwise. Then the operator norm $\|\frac{\partial}{\partial\Sigma}x'_{s_j}VX'Y\|$ is $\mathcal{O}_P\left(1\right)$.

(1) leads to

$$\left\|\frac{\partial}{\partial\Sigma}\Delta_j\right\| = \mathcal{O}\left(n_j/\phi^2\right)\left[\mathcal{O}\left(1\right) + \mathcal{O}_P\left(\phi^2/n_j\right) + \mathcal{O}_P\left(\phi/\sqrt{n_j}\right)\right] = \mathcal{O}_P\left(n_j\right),$$

and

$$N^{-1}\left\|\frac{\partial}{\partial\Sigma}\Delta_j\right\| = \mathcal{O}_P\left(n_j/N\right).$$

A similar argument to the above for $\phi$ holds if $\left\|\frac{\partial}{\partial\phi}V\right\| = \mathcal{O}_P\left(1\right)$. Let $M \in I\!R^{P\times P}$ be a binary matrix where $M_{pq} = 1$ if $\phi^{-2}(X'X)_{pq} \neq 0$. Then:

$$\frac{\partial}{\partial\phi}x'_{s_j}Vx_{s_j} = -\frac{2}{\phi^3}x'_{s_j}VMVx_{s_j}.$$

It can be seen that the absolute value of this scalar quantity is non-increasing as $J$ increases from Lemma A.1, item 2. Also see Remark 1 of Lemma A.1.

We established that the operator norm of the difference in the derivatives for $N^{-1}(\ell(\phi,\Sigma|Y_{-s_j}) - \ell(\phi,\Sigma|Y))$ is $\mathcal{O}_P\left(n_j/N\right)$. Note that $\operatorname{argmax}_{\Sigma,\phi}\ell(\phi,\Sigma|Y)$ is the inverse of the gradient for $N^{-1}\ell(\phi,\Sigma|Y)$ when the latter is 0. Then the rate at which $N^{-1}\ell(\phi,\Sigma|Y_{-s_j}) - N^{-1}\ell(\phi,\Sigma|Y) \to 0$ is the rate at which $\operatorname{argmax}_{\Sigma,\phi}\ell(\phi,\Sigma|Y) - \operatorname{argmax}_{\Sigma,\phi}\ell(\phi,\Sigma|Y_{s_j}) \to 0$ if $N^{-1}\Delta_j$ is uniformly continuous and bijective. Using the same methods as determining $\frac{\partial}{\partial\Sigma}\Delta_j$, it can be shown that $\frac{\partial}{\partial\Sigma}\Delta_j$ is differentiable. Note that $\phi$ is a positive scalar following a proper prior in our setting. While it can theoretically attain a zero value, the probability of exactly zero occurring is negligible. Thus, $\phi$ is $\mathcal{O}_P\left(1\right)$, $\phi^{-1}$ is also $\mathcal{O}_P\left(1\right)$, and $\|XVX'\| \leq \phi^2 = \mathcal{O}_P\left(1\right)$

from the definition of $V$. Note that the randomness for $\phi$ and $\phi^{-1}$ come from their prior distributions following the Bayesian point of view. Hence the operator norm of the derivative is finite in probability, thus $\frac{\partial}{\partial \Sigma}\Delta_j$ is uniformly continuous in probability. Proof by contradiction shows that $\frac{\partial}{\partial \Sigma}\Delta_j$ is a bijective function. In fact, suppose there are $\Sigma_1$ and $\Sigma_2$ such that $\Sigma_1 \neq \Sigma_2$ and $\frac{\partial}{\partial \Sigma}\Delta_j$ corresponding to $\Sigma_1$ and $\Sigma_2$ are equal. Then from the mean value theorem, we have $\Sigma_0$ such that $\frac{\partial^2}{\partial \Sigma^2}\Delta_j|_{\Sigma=\Sigma_0} = 0$, which means that $\frac{\partial^2}{\partial \Sigma_{s_k,l}\partial \Sigma}\Delta_j|_{\Sigma=\Sigma_0} = 0$ for any $k$ and $l$. Then we have

$$\frac{n_j/\phi^2}{1-\frac{n_j}{\phi^2}x'_{s_j}Vx_{s_j}}\left(\frac{1}{2}\left(1+\frac{\bar{e}_{s_j}^2}{1-\frac{n_j}{\phi^2}x'_{s_j}Vx_{s_j}}\right)\frac{\partial^2}{\partial \Sigma_{s_k,l}\partial \Sigma}x'_{s_j}Vx_{s_j}+\bar{e}_{s_j}\frac{\partial^2}{\partial \Sigma_{s_k,l}\partial \Sigma}x'_{s_j}VX'Y\right)=0, \tag{2}$$

when $\Sigma = \Sigma_0$ for any $k$ and $l$, since these terms on the left-hand side of (2) are the only ones in $\frac{\partial^2}{\partial \Sigma_{s_k,l}\partial \Sigma}\Delta_j|_{\Sigma=\Sigma_0}$ whose column spaces and row spaces are varying with $l$ and $k$, respectively. Furthermore, calculating (2) with Lemma A.3 and rearranging terms, we can see that (2) holds for any $k$ and $l$ if and only if $X'Y = \frac{1}{2}\left(\frac{1}{\bar{e}_{s_j}}+\frac{\bar{e}_{s_j}}{1-\frac{n_j}{\phi^2}x'_{s_j}Vx_{s_j}}\right)x_{s_j}$ when $\Sigma = \Sigma_0$. Hence, we have

$$\frac{\partial^2}{\partial \Sigma_{s_k,l}\partial \Sigma}\Delta_j|_{\Sigma=\Sigma_0} = \frac{n_j^2\bar{e}_{s_j}^2/\phi^4}{2(1-\frac{n_j}{\phi^2}x'_{s_j}Vx_{s_j})^3}\frac{\partial}{\partial \Sigma_{s_k,l}}\left(x'_{s_j}Vx_{s_j}\right)\frac{\partial}{\partial \Sigma}\left(x'_{s_j}Vx_{s_j}\right)=0,$$

which cannot be true simultaneously for any $k$ and $l$. Then we have shown that $\frac{\partial}{\partial \Sigma}\Delta_j$ is a bijective function by contradiction. Thus we have completed the proof of the theorem. □

We re-state Corollary 2.2 below:

Let $\hat{\Sigma}$ denote the full-data posterior mean, $E[\Sigma|Y]$, and $\tilde{\Sigma}$ the CV posterior mean over the training data $E[\Sigma|Y_{-s_j}]$ for CV fold $j$. Note that $X_{s_j}$ is made up of identical rows, let $x'_{s_j}$ refer to any row in $X_{s_j}$. Under the same conditions as Theorem 2.1, $E[x'_{s_j}\beta|Y_{-s_j}] = E[x'_{s_j}\beta|Y_{-s_j},\hat{\Sigma},\hat{\phi}](1+\mathcal{O}_P(n_j/N))$ as $J$ goes to infinity.

*Proof.* From equation (3.7) in (Kass and Steffey, 1989), we can approximate $E[x'_{s_j}\beta|Y_{-s_j}]$ with $E[x'_{s_j}\beta|Y_{-s_j},\Sigma,\phi]$ using the cross-validated posterior means of $\Sigma$ and $\phi$, $E[\Sigma|Y_{-s_j}]$ and $E[\phi|Y_{-s_j}]$, respectively. This approximation has error $\mathcal{O}((N-n_j)^{-1})$, which furthermore is of order $\mathcal{O}(n_j/N)$.

The analytical form for $E[x'_{s_j}\beta|Y_{-s_j}]$ is in (3). Substituting $E[\Sigma|Y_{-s_j}] = \hat{\Sigma}(1+\mathcal{O}_P\left(n_j/N\right))$, where the $\mathcal{O}_P$ notation for matrices means that the operator norm of the corresponding matrix has the specified $\mathcal{O}_P$ bound, and $E[\phi|Y_{-s_j}] = \hat{\phi}(1 + \mathcal{O}_P\left(n_j/N\right))$ into (3) and an application of Sherman-Morrison yields

$$\phi^{-2}XVX'Y + \mathcal{O}_P\left(n_j/N\right)XVX'XVX'Y(\phi^{-2} + \mathcal{O}_P\left(n_j/N\right)) + \mathcal{O}_P\left(n_j/N\right)XVB\Sigma^{-1}B'VX'Y,$$

where $B \in I\!R^{P\times P_2}$ is a block matrix of 0s in the first $P_1$ rows and the identity matrix in the remaining $P_2$. As the operator norms $\|XVX'\|, \|XVX'Y\|, \|\Sigma\|$ are all $\mathcal{O}_P\left(1\right)$, the result follows. □

# B Review of existing CV approximation methods

We provide a brief review of existing CV approximation methods: ghosting (GHOST) (Marshall and Spiegelhalter, 2003), integrated importance sampling (iIS) (Li et al., 2016; Vanhatalo et al., 2013; Vehtari et al., 2016), and likelihood-based linear approximations (Giordano et al., 2019; Jaeckel, 1972; Rad and Maleki, 2020).

## B.1 Ghosting

Ghosting draws $\tilde{\theta}_j^{(m)}$ from $f(\theta_j|\theta_{-j}^{(m)}, \beta_{/\theta}^{(m)}, \Sigma^{(m)}, Y)$ for each posterior sample $m$. The $M$ total "ghost" samples are then used as an approximation of $f(\theta_j|Y_{-s_j})$. If $\theta_j$'s are independent given the variance hyperparameters, $\Sigma$, then the ghost samples $\tilde{\theta}_j^{(m)}$ are simply drawn from $\theta_j|\Sigma^{(m)}$. This mimics the effect of treating the held-out test data $Y_{s_j}$ as an unknown cluster. The training data posterior densities for $\beta_{/\theta}$ and $\Sigma$ are approximated by the full-data posterior densities. The ghosting estimate is then $E[X_{\beta_{/\theta}}\beta_{/\theta}|Y] + E[X_\theta\theta_j|\theta_{-j}, \Sigma, \beta_{/\theta}, Y]$. Note that the Ghosting is only applicable to the LMM cases.

## B.2 Integrated importance sampling (iIS)

iIS methods integrate out the cluster-specific effects $\theta_j$ using the importance sampling weights of Gelfand et al. (1992). Importance sampling (IS) approximates the target training

data posterior density $\beta, \phi$ by re-weighting full-data posterior samples. The weights for the $j^{th}$ CV fold and $m^{th}$ posterior sample, $w_j^{\text{IS}}(m)$, are proportional to a ratio of the two densities,

$$w_j^{\text{IS}}(m) = \frac{1}{f(Y_{s_j}|\phi^{(m)}, \beta^{(m)}, \Sigma^{(m)})} = \frac{1}{f(Y_{s_j}|\phi^{(m)}, \beta^{(m)}, \Sigma^{(m)}, Y_{-s_j})} \propto \frac{f(\beta^{(m)}, \phi^{(m)}, \Sigma^{(m)}|Y_{-s_j})}{f(\beta^{(m)}, \phi^{(m)}, \Sigma^{(m)}|Y)}. \tag{3}$$

The equality follows from the independence of the $Y_{s_j}$'s given $\beta$ and $\phi$. Mean estimates are obtained by averaging over the posterior samples, weighted by $w_j^{(m)}$,

$$\hat{Y}_{s_j}^{\text{IS}} = \left( \sum_{m=1}^{M} w_j^{\text{IS}}(m) \right)^{-1} \sum_{m=1}^{M} X\beta^{(m)} w_j^{\text{IS}}(m).$$

Dividing over the sum of $w_j^{\text{IS}}$'s is a correction for knowing the ratio only up to a constant (Gelfand, 1996).

IS is asymptotically unbiased for LCO-CV as the number of MC samples, $M$, increases, but it can be a poor approximation in practice when the importance sampling weights have a large variance (Li et al., 2016; Merkle et al., 2019). In extreme cases, large importance sampling weights on only a few points may dominate the estimate, leading to an unreliable estimate (Owen, 2013). In practice, IS is most often used with leave-one-out CV (LOO-CV), when the difference between densities is relatively small.

iIS addresses this issue by integrating out $\theta_j$ but still conditioning on the rest of the parameters, $\theta_{-j}^{(m)}$ and $\beta_{/\theta}^{(m)}$, that are less impacted by the removal of $Y_{s_j}$. The weights and estimates under iIS are presented below and their derivations are included in the computational complexity calculations of Appendix C.

$$w_j^{\text{iIS}}(m) = \frac{1}{f(Y_{s_j}|\phi^{(m)}, \Sigma^{(m)}, \theta_{-j}^{(m)}, \beta_{/\theta}^{(m)})}, \quad \hat{Y}_{s_j}^{\text{iIS}} = \frac{\sum_{m=1}^{M} w_j^{\text{iIS}}(m) E[Y_{s_j}|\phi^{(m)}, \Sigma^{(m)}, \theta_{-j}^{(m)}, \beta_{/\theta}^{(m)}]}{\sum_{m=1}^{M} w_j^{\text{iIS}}(m)}, \tag{4}$$

We used Pareto-smoothed importance sampling (PSIS, Vehtari et al. (2015), `R` package `loo`) to stabilize the importance weights and ensure finite variance for iIS method. Although the method originally applies to the IS weights in (3), Pareto-smoothing can be applied to all importance sampling weights.

## B.3 Vehtari's approximation

Another Bayesian LOO-CV approximation method is proposed by Vehtari et al. (2016). It considers Gaussian latent variable models where the integration over the latent variables is approximated using the Laplace method or expectation propagation. We adopt the key idea of Vehtari's approximation and further extend it to LCO-CV approximation.

In LCO-CV, the latent variable to be integrated is $\theta_j$, the random effects that are associated with the test data, $Y_{s_j}$. As suggested by Vehtari et al. (2016), we first assume that removing $Y_{s_j}$ has only a small impact on the posterior distribution of $(\beta_{/\theta}, \phi, \Sigma)$, the set of parameters that do not pertain to the CV design. The following steps are used to approximate the predictive distribution of $Y_{s_j}|Y_{-s_j}$:

1. Use the Laplace method to approximate the conditional posterior distribution of $\theta_j$ given the training data: $f(\theta_j|\beta_{/\theta}^{(m)}, \phi^{(m)}, \Sigma^{(m)}, Y_{-s_j})$, where $m$ indicates the $m$th Monte Carlo sample for the full data posterior.

2. Since the predictive distribution of $Y_{s_j}$ conditional on all parameters is easy to compute, we combine it with the conditional posterior of $\theta_j$ from Step 1 and integrate out $\theta_j$. Thus, for each $m$, we compute $Y_{s_j}^{(m)} = E(Y_{s_j}|\beta_{/\theta}^{(m)}, \phi^{(m)}, \Sigma^{(m)})$.

3. The LCO estimate of $Y_{s_j}$ is then $\hat{E}(Y_{s_j}|Y_{-s_j}) = \frac{1}{M}\sum_{m=1}^{M} Y_{s_j}^{(m)}$.

## B.4 Likelihood-based linear approximations (NS and IJ)

Likelihood-based linear approximations can also be used to obtain the maximum likelihood estimate (MLE) or the maximum a posteriori probability (MAP) (MAP, or posterior mode) estimate under cross-validation. The posterior mode can be estimated using computationally-efficient optimization methods and approaches the posterior mean as the number of observations $N \to \infty$, making it attractive in situations with large amounts of data. We compared AXE to two approaches that fall into this category, Newton-Raphson (NS) (Rad and Maleki, 2020; Wang et al., 2018) and the infinitesimal jackknife (IJ) (Giordano et al., 2019). For both methods, we use the automated differentiation software package `autograd` (Maclaurin et al., 2015) to obtain gradients and Hessians.

NS uses the full-data posterior mode for all parameters as a starting value and then takes a single Newton-Raphson step to approximate the training data posterior mode. Let $\Xi$ denote the set of all model parameters $\Xi := \{\beta, \Sigma, \phi\}$; let $\ell(Y, \Xi)$ denote the log joint posterior density of $\Xi$ and $Y$, $\ell(Y, \Xi) := \sum_i \log f(Y_i|\Xi) + \log f(\Xi)$; let $\dot{\Xi}$ denote the posterior mode given the full data, $\dot{\Xi} = \text{argmax}_{\Xi}\, \ell(Y, \Xi)$; and let $\dot{\Xi}_{-j}$ denote the posterior mode given the training data. Then the NS approximate for $\dot{\Xi}_{-j}$ is

$$\tilde{\Xi}^{\text{NS}}_{-j} = \dot{\Xi} - \left[\nabla^2 \ell(Y_{-s_j}, \Xi)\right]^{-1} \nabla \ell(Y_{-s_j}, \Xi)\Big|_{\Xi=\dot{\Xi}}. \tag{5}$$

Note that, under LCO-CV, the Hessian in (5) is singular, as $\frac{\partial}{\partial \theta_j}\ell(Y_{-s_j}, \Xi) = 0$. We adapt NS in two ways for LCO-CV: 1) we omit $\theta_j$ from the set of model parameters, using $\Xi_{/\theta_j}$ in all instances rather than $\Xi$; 2) we integrate out all $\theta$ and reduce the parameter space to $\Xi_{/\theta} = \{\beta_{/\theta}, \Sigma, \phi\}$. We refer to the former as NS-C for conditioning on $\Xi_{/\theta_j}$ and the latter as NS-A for integrating over all $\theta$. NS-A accounts for the known change in $\theta_j$ across CV folds and reduces the number of parameters, which speeds up computation. In most cases, NS-C produced worse estimates for our examples, likely because the change in $\theta_j$ led to large gradient values, causing the linear approximation to be less accurate.

In general, NS requires re-calculating the Hessian and its inverse for each CV fold, which can be computationally expensive. If $Y_i|\Xi$ are independent across $i$, where $i$ denotes individual observations, $i = 1, \ldots, N$ and $Y \in I\!R^N$, and the $Y_i$ share the same mean $x'_{s_j}\beta$, then the Hessian in (5) may be re-written as $\nabla^2 \log f(Y|\Xi) - \sum_{i \in s_j} \nabla^2 \log f(Y_i|\Xi)$. Then $\nabla^2 \log f(Y_i|\Xi)$ is some scalar times $x_{s_j}x'_{s_j}$, a rank-one matrix, and the inverse can be calculated analytically with Sherman-Morrison which can reduce the amount of computation (Stephenson and Broderick, 2020, Section D.5). Rad and Maleki (2020) present an alternative form for (5) which requires calculating the Hessian only once, but their adjustment applies only to LOO-CV. For LOO-CV, Theorem 6 of Rad and Maleki (2020) states that under certain conditions, the mean error for NS is o(1/N).

IJ was originally developed by Jaeckel (1972) and has recently been adapted for cross-validation (Beirami et al., 2017; Giordano et al., 2019; Koh et al., 2019). If $Y_i|\Xi$ are independent across $i$, then the estimation of $\Xi$ in the cross-validation setting can be con-

ceptualized as

$$\dot{\Xi}(w) = \underset{\Xi}{\text{argmax}} \sum_{i=1}^{N} w_i \log f(Y_i|\Xi) + \log f(\Xi), \tag{6}$$

where $w_i = 1$ for observations in the training data and $w_i = 0$ for observations in the test data. When $w = \mathbb{1}_N$, $\dot{\Xi}(w)$ is the full-data posterior mode, denoted as $\dot{\Xi}$. We obtain the cross-validated posterior mode $\dot{\Xi}_{-j}$ by setting $w_i$ to 0 for all $i \in s_j$. Let $\ell(Y, w, \Xi)$ be the joint density given weight vector $w$, $\ell(Y, w, \Xi) := \sum_{i=1}^{N} w_i \log f(Y_i|\Xi) + \log f(\Xi)$. IJ constructs a first-order Taylor series expansion of $\Xi(w)$ around $w = \mathbb{1}_N$ to estimate $\Xi_{-j}$, yielding

$$\dot{\Xi}^{\text{IJ}}_{-j} = \dot{\Xi} - \sum_{i \in s_j} \left[\nabla^2 \ell(Y, w, \Xi)\right]^{-1} \frac{\partial}{\partial w_i} \nabla \ell(Y, w, \Xi)\Big|_{\Xi=\dot{\Xi} \text{ and } w=\mathbb{1}_N}, \tag{7}$$

where the gradient and the Hessian are taken with respect to $\Xi$. Similar to NS, we compare two versions of IJ. IJ-A integrates out $\theta$ and IJ-C retains all model parameters except $\theta_j$. In general, the Hessian under IJ can be calculated just once, in comparison to NS where the Hessian is re-calculated for every CV fold. Note that the Hessian is the summation of $N$ individual Hessians, $\nabla^2 \ell(Y, w, \Xi) = \sum_{i=1}^{N} \nabla^2 \ell(Y_i, w, \Xi)$. Theorem 1 of Giordano et al. (2019) shows that when specific assumptions are met, including the sample variances of the gradients and Hessians being uniformly bounded and their summation over $i \in s_j$ likewise being uniformly bounded for all CV folds $j$, then the error for IJ is low.

As written in (7), IJ requires $Y_i|\Xi$ to be independent across $i$. When the $\theta$ are dependent, so are the $Y_i|\Xi$ and the factorization in (6) is no longer available. Ghosh et al. (2020) provide an extension of IJ called structured IJ which can be used for dependent $\theta$ by integrating it out. However, the integration is not a trivial task.

# C Computational complexity calculations

This section contains derivations for computational complexity in Table 2. We assume without loss of generality that $P_2 \asymp P$, which means that $P_2$ and $P$ are of the same order, as $P_2 \leq P$ and the number of fixed effects $P_1$ is often small. For all methods, we assume that the cost of drawing a sample from a specific density is dominated by calculating the

density's parameters, e.g., if drawing from a multivariate normal density, we assume the calculation of the mean vector and covariance matrix dominates the computational cost.

## C.1 AXE

We re-state the AXE estimate below:

$$\hat{Y}_{s_j}^{AXE} = \frac{1}{\hat{\phi}^2} X_{s_j} \left( \frac{1}{\hat{\phi}^2} X'_{-s_j} X_{-s_j} + \begin{bmatrix} 0 & 0 \\ 0 & \Sigma^{-1} \end{bmatrix} \right)^{-1} X'_{-s_j} Y_{-s_j}. \tag{8}$$

The cost of the matrix inversion in (8) is $\mathcal{O}(P^3)$, while the matrix multiplication is $\mathcal{O}(n_j N P^2)$. Conducted over $J$ total cross-validation loops, the computational complexity for AXE is $\mathcal{O}(N^2P^2 + JP^3))$.

## C.2 GHOST

Without loss of generality, let $f(\theta|\Sigma)$ be $N(0, \Sigma)$. Then:

$$\theta_j|\theta_{-j}, \Sigma \sim N(\Sigma_{j-j}\Sigma_{-j-j}^{-1}\theta_{-j}, \quad \Sigma_{jj} - \Sigma_{j-j}\Sigma_{-j-j}^{-1}\Sigma_{-jj}). \tag{9}$$

As we assume the dimension of $\theta_j$ is fixed, the cost of the matrix inversion in (9) is $\mathcal{O}(P^3)$ and the cost of the matrix multiplication is likewise $\mathcal{O}(P^3)$, which is repeated for all $J$ cross-validation loops and $M$ samples. Once $\hat{\theta}^{(\text{GHOST})}$ is obtained, the ghosting estimate

$$\hat{Y} = X_{\beta_{/\theta}}\beta_{/\theta} + X_\theta\hat{\theta}^{(\text{GHOST})},$$

has cost $\mathcal{O}(N)$. The total computational complexity for GHOST is $\mathcal{O}(MJP^3 + N)$.

## C.3 iIS

We re-state the iIS importance weights $w_j^{(m)}$ and mean estimate for $E[Y_{s_j}|Y_{-s_j}]$:

$$w_j^{(m)} = \frac{1}{f(Y_{s_j}|\theta_{-j}^{(m)}, \phi^{(m)}, \beta_{/\theta}^{(m)}, \Sigma^{(m)})}, \quad \hat{Y}_{s_j} = \frac{\sum_{m=1}^M w_j^{(m)} E\left[Y_{s_j}|\theta_{-j}, \phi^{(m)}, \Sigma^{(m)}, \beta_{/\theta}^{(m)}\right]}{\sum_{m=1}^M w_j^{(m)}}. \tag{10}$$

Let $a_{s_j} := E[\theta_j|\theta_{-j}, \Sigma]$ and $D := \text{Cov}(\theta_j|\theta_{-j}, \Sigma)$, corresponding to the mean and covariance, respectively, of (9). Then,

$$Y_{s_j}|\theta_{-j}, \phi, \beta_{/\theta}, \Sigma \sim N(X_{\beta_{/\theta}, s_j}\beta_{/\theta} + a_{s_j}, \phi^2 I + D).$$

Note that the cost of obtaining $a_{s_j}$ and $D$ for all CV folds and MC samples is the same as ghosting at $\mathcal{O}(MJP^3)$. The cost of obtaining the likelihood in weight $w_j^{(m)}$ for (10) is an additional $\mathcal{O}\left(M\sum_{j=1}^J n_j\right) = \mathcal{O}(MN)$. The cost of $X_{\beta_{/\theta}, s_j}\beta_{/\theta} + a_{s_j}$ over all CV folds $J$ is $\mathcal{O}(N)$. The total cost is then $\mathcal{O}(MJP^3 + MN)$.

## C.4 Vehtari

Similar to iIS, drawing from this density is equivalent to running AXE for every MC sample $s$ and has computational cost $\mathcal{O}(M(N^2P^2 + JP^3))$ across all CV folds and MC samples. Obtaining the likelihood in $f(Y_{s_j}|\phi^{(m)}, \Sigma^{(m)}, Y_{-s_j})$ has additional computational cost $\mathcal{O}(MJN)$. As $n_j \geq 1$ for all $J$, $J$ is $\mathcal{O}(N)$, this additional cost is $\mathcal{O}(MN^2)$. The total computational cost is then $\mathcal{O}(M(N^2P^2 + JP^3))$.

## C.5 IJ-A

Computational costs for IJ-A were calculated in Ghosh et al. (2020) and are included here for completeness. We re-state the IJ-A approximate, which is

$$\dot{\Xi}_{-j}^{\text{IJ-A}} = \dot{\Xi} - \sum_{i \in s_j} \left[\nabla^2 \ell(Y, w, \Xi)\right]^{-1} \frac{\partial}{\partial w_i} \nabla \ell(Y, w, \Xi)\Big|_{\Xi = \dot{\Xi}, w = \mathbb{1}_N}.$$

Inversion of the Hessian occurs once across all CV folds and thus incurs cost $\mathcal{O}(P_\Xi^3)$ where $P_\Xi$ is the dimension of $\Xi$. Multiplication of the Hessian and the gradient vector occurs for each of the $N$ data points and incurs cost $\mathcal{O}(P_\Xi^2 N)$. Calculation of the Hessian and gradients is performed using the automatic differentiation module `autograd` (Maclaurin et al., 2015), which Bartholomew-Biggs et al. (2000) showed requires the same computation, up to a constant, as calculation of $\ell(Y, w, \Xi)$ itself. The cost of obtaining $\hat{Y}^{\text{IJ-A}}$ has cost $\mathcal{O}(NP)$. The total computational cost is then $\mathcal{O}(P_\Xi^3 + P_\Xi^2 N + NP)$.

### C.6 NS-A

We re-state the NS-A approximate, which is

$$\dot{\Xi}^{\text{NS-A}}_{-j} = \dot{\Xi} - \left[\nabla^2 \ell(Y_{-s_j}, \Xi)\right]^{-1} \nabla \ell(Y_{-s_j}, \Xi)\Big|_{\Xi=\dot{\Xi}}.$$

For each of the $J$ CV folds, inversion of the Hessian and subsequent multiplication by the gradient has computational cost of $\mathcal{O}\left(P_\Xi^3 + P_\Xi^2\right)$, where $P_\Xi$ is the dimension of $\Xi$. Calculation of the Hessian and gradients are performed using the automatic differentiation module `autograd` (Maclaurin et al., 2015), which Bartholomew-Biggs et al. (2000) showed requires the same computation, up to a constant, as calculation of $\ell(Y_{-s_j}, \Xi)$ itself. The cost of obtaining $\hat{Y}^{\text{NS-A}}$ has cost $\mathcal{O}(NP)$. The total computational cost is then $\mathcal{O}\left(JP_\Xi^3 + NP\right)$.

### C.7 MCV (Gibbs sampling)

Under the model in (1) with prior densities $\Sigma \sim IW(\nu, \Psi)$, $\phi \sim \Gamma^{-1}(a, b)$, $\beta_1 \sim N(0, C)$, $\beta_2|\Sigma \sim N(0, \Sigma)$, one Gibbs sampling scheme is as follows:

$$\beta^{(m)}|\Sigma^{(m-1)}, \phi^{(m-1)}, Y \sim N(\phi^{-2(m-1)} V^{(m-1)} X^T Y, V^{(m-1)}), \tag{11}$$

$$V^{(m-1)} = (\Sigma^{-1(m-1)} + \frac{1}{\phi^{2(m-1)}} X^T X)^{-1}$$

$$\Sigma^{(m)}|\beta^{(m)}, \phi^{(m-1)}, Y \sim IW(N + v, \Psi + (\beta_2^{(m)} - X_{\beta_{/\theta}} \beta_{/\theta})(\beta_2^{(m)} - X_{\beta_{/\theta}} \beta_{/\theta})^T) \tag{12}$$

$$\phi^{2(m)}|\Sigma^{(m)}, \beta^{(m)}, Y \sim \Gamma^{-1}(a + \frac{1}{2}N, b + \frac{1}{2}(Y - X\beta^{(m)})^T (Y - X\beta^{(m)}), \tag{13}$$

where $m$ refers to the $m$th iteration of the Gibbs sampler, $IW$ refers to the inverse-Wishart distribution and $\Gamma^{-1}$ the inverse-gamma.

We assume that the cost of drawing a sample from the specified densities is dominated by the re-calculation of parameters within each iteration. For example, the cost of drawing $\beta^{(m)}$ is dominated by the calculations of $V^{(m-1)}$ and $V^{(m-1)} X^T Y$.

In eq. (11), the inversion of $V$ is $\mathcal{O}(P^3)$, while the multiplication of $VX'Y$ is $\mathcal{O}(NP^2)$. The cost of eq. (12) is $\mathcal{O}(P_2^2)$. The cost of eq. (13) is $\mathcal{O}(N^2 P)$. The cost of obtaining $\hat{Y}$ has cost $\mathcal{O}(NP)$. For each iteration of the Gibbs sampler, the computational cost is $\mathcal{O}(P^3 + NP^2 + N^2 P)$. Then with $M$ iterations of the Gibbs sampler, the computational complexity is $O(MN^2 P + MNP^2 + MP^3)$.

# D Example data sets and models

This section describes each of the data sets and models in detail, with all results compiled and described together in Section 4.

## D.1 Eight schools

The eight schools data comes from a meta-analysis conducted by Rubin (1981) on the effects of coaching on verbal SAT scores and appears frequently in the literature. The data consist of mean $Y_{s_j}$ and standard error $t_j$ of treatment effects from school $j$, with a total of eight schools. As $n_j = 1$ for all $j$, we denote $Y_{s_j}$ as simply $Y_j$ and $t_j$ as $t_j$. The data are modeled as a one-way linear mixed effects model,

$$Y_j \sim N(\mu + \theta_j, t_j^2), \quad \theta_j \sim N(0, \sigma^2), f(\mu) \propto 1, f(\sigma) \propto 1$$

where $\mu$ and $\sigma$ are scalar values with improper uniform priors. In this one-way model, $\beta_{/\theta} = \beta_1 = \mu$, while $\theta_j$ corresponds to $\beta_2$.

We re-create a scenario derived from Vehtari et al. (2016), where the eight $Y_j$ are multiplied by a data scaling factor $\alpha$—since $t_j$ are given and fixed as part of the data, this has the effect of increasing the variance $\Sigma$ and decreasing the amount of data pooling. The model then becomes:

$$\alpha Y_j \sim N(\alpha\mu + \alpha\theta_j, t_j^2), \quad \alpha\theta_j \sim N(0, \alpha^2\sigma^2), f(\mu) \propto 1, f(\alpha\sigma) \propto 1.$$

For each scaling factor $\alpha \in \{0.1, 0.2, \ldots, 3.9, 4.0\}$, cross-validation is conducted by withholding each of the $Y_j$ in turn. The CV design is then both LCO-CV and LOO-CV because we observe one mean estimate per school. We fit the models in STAN, running four chains of 2000 samples each, with the first 1000 as burn-in. MC diagnostics indicated that the chains may not have converged for three of the $\alpha$ values $(2.2, 2.4, 2.8)$, and we increased the number of burn-in to 4000 for 5000 total samples.

## D.2 Radon

The Radon data measures the log radon level of 919 houses in Minnesota and contains data on location (`county`), the level of uranium in the county (`log uranium`), and whether the house contains a basement (`basement`). It is included as part of the `rstanarm` package (Goodrich et al., 2018) via Gelman and Hill (2007).

We examine three models where all three define response vector $Y$ as the log radon level of the house and the `county` covariate as a random effect with `county` $\sim N(0, \sigma^2)$, while all other effects are fixed effects,

$$\text{Model 1: } Y \sim N(a_0 + \texttt{county}, \phi^2),$$
$$\text{Model 2: } Y \sim N(\texttt{basement} + \texttt{county}, \phi^2),$$
$$\text{Model 3: } Y \sim N(\texttt{basement} + \log \texttt{uranium} + \texttt{county}, \phi^2).$$

Models were fit using the `rstanarm` package Goodrich et al. (2018), using the default priors for `stan_lmer`. Cross-validation was performed over counties, with each loop removing all houses within one county as test data. Using the notation from (7), $\theta$ corresponds to the `county` random effect and $\beta_{/\theta}$ corresponds to the fixed effects, which is $a_0$ for Model 1, `basement` for Model 2, and `basement` + `log uranium` for Model 3. There are 85 counties in the data with a median of 5 houses per county. Two of the counties contain data on over 100 houses, each making up over 11% of the data.

## D.3 Radon subsets

The Radon subsets are a set of simulations where the test data are fixed as the 23 samples from the county of Olmsted. The training data are a randomly selected subset of counties such that the total number of counties $J$ is $\{3, 4, 6, 9, 12\}$ (including Olmsted) and the training data size is approximately $\{77, 58, 46, 38, 32\}$, which corresponds to approximate test data proportions $\delta = \{0.3, 0.4, 0.5, 0.6, 0.7\}$. For all combinations of $J$ and $\delta$, we derive the AXE and MCV values for at most 60 different iterations of training data (for $J = 3$ and $\delta = 0.3$, there are only 35 iterations available due to the data availability), using the

three models in subsection D.2. Models were again fit using the `rstanarm` package with the default priors for `stan_lmer`.

The data imbalance among counties can lead to certain counties being over-represented across the sets of training data. To mitigate this, we restrict each training data set to have a unique combination of county sizes. The combinations are such that they are within 10% of the target training data size $n_{\text{target}} \coloneqq 23(1-\delta)$, meaning that $n_{-i}$ is within $n_{\text{target}} \pm 0.1 n_{\text{target}}$. The sample is inversely weighted by how far the training data set size is from $n_{\text{target}}$. Within each of the 60 combinations, all matching counties are found, and one final combination is sampled.

Model 3 contains both the fixed `floor` effect and the continuous fixed effect for `log uranium`, which is defined at the county level only. For the full Radon data, that means there are 84 unique values of `log uranium` in the training data; for the Radon subsets data, the number of unique values ranges from 2 to 11.

## D.4 Esports players (ESP)

The Esports player data consists of professional player statistics from the popular video game "League of Legends" for players in the North American League Championship Series from January 2020 - June 2020. In a game, two teams of five players compete to capture the opposing team's base. The data include the player's name (`player`); the player's team name (`team`); the player's position on the team (`position`); the name of the player's character in-game (`champion`); log earned gold per minute (`log_egpm`, continuous); log damage per minute (`log_dpm`, continuous), and the player's kills in the game (`Y`). There are 73 unique players, ten unique teams, five unique positions, and 108 unique champions. The data are publicly available at `oracleselixir.com`, which also contains many other in-game statistics.

We model the number of kills a player $p$ achieves in a game $g$ on champion $c$ as a Poisson GLMM. Let design matrix $X$ corresponding to the fixed effects consist of a vector of 1's, binary indicator vectors for `team`, binary indicator vectors for `position`, `log_dpm`, and

log_egpm, and let $X_{pgc}$ correspond to the row in $X$ with player $p$, game $g$, and champion $c$:

$$Y_{pgc} \sim \text{Poisson}(\eta_p)$$
$$\log(\eta_{pgc}) = X_{pgc}\boldsymbol{a} + \alpha_p + \alpha_c,$$

where $\boldsymbol{a}$ is the vector of fixed effect coefficients, $\alpha_p$ corresponds to a player-specific random intercept, and $\alpha_c$ is a champion-specific random intercept which is crossed with players. Players are typically nested within position and team, although 5 players within the data set are represented with more than one team.

The model was fit in rstanarm using default priors for stan_glmer. Cross-validation folds are defined by the player-specific random intercept $\alpha_p$. Using the notation in (7), $\beta_{/\theta}$ corresponds to $(\boldsymbol{a}, \alpha_c)$ and $\theta$ corresponds to $\alpha_p$. There are 73 total players with a median number of 33 games within the data is 33, a minimum of 2, and a maximum of 56. The AXE approximation is as described in Section 2.2, where $\tilde{Y} = \log\big(E[Y_{s_j}|Y]\big)$ and $\Phi = \text{diag}(1/E[X\beta|Y])$.

## D.5 Scottish Lip Cancer (SLC)

The Scottish Lip Cancer data consist of total observed male lip cancer counts collected over the time period 1975-1980 in $J = 56$ districts of Scotland; the number of expected cases, $E_j$, calculated based on standardization of "population at risk" across different age groups; the percent of population employed in agriculture and forestry $\mathrm{d}_j$; and an adjacency matrix $A$, where $A_{jj} = 0$, $A_{ji} = 1$ if $j$ and $i$ are neighboring districts, and $A_{ji} = 0$ otherwise. It is available through the CARBayesST package (Lee et al., 2018) in R.

The cross-validation folds are defined by the istricts, thus $n_j = 1$ for all $j = 1, \ldots, J$ and we denote $\mathcal{S}_|$ simply as $j$. The number of expected cases $E_j$ is used as an offset in a Poisson GLMM of male lip cancer counts where

$$Y_j|\eta_j, E_j \sim \text{Poisson}(\eta_j E_j)$$

and $\log(\eta_j)$ contains the fixed and random effects of the GLMM. In this scenario, the fixed effects consist of a grand mean intercept and term linear in $d_j$. The random effects consist

of district-level random intercepts, which are modeled such that each $\theta_j$ is dependent on the values of its neighbors:

$$\theta_j|\theta_{-j} \sim N(\rho_j \sum_{i=1}^{n} A_{ji}\theta_i, \sigma^2), \quad \rho_j \in [0,1]$$

$$\log(\eta_j) = a_0 + a_1 \mathrm{d}_j + \theta_j.$$

The value of $\rho_j$ controls the spatial dependence among neighboring districts, where 0 indicates no spatial dependence. The joint distribution of the $\theta_j$ simplifies to:

$$\theta_j \sim N(0, \sigma^2(\mathrm{diag}(A\mathbb{1}) - \rho_j A)^{-1}).$$

The covariance of the $\theta_j$ is a simplified version of a proper conditional auto-regression (CAR). As the $\theta_j$, given neighboring $\theta_k$, are independent of all remaining $\theta$, this is also a Gaussian Markov random field.

As the cross-validation folds are defined by the districts, LCO-CV is equivalent to LOO-CV. $\theta$ as defined in the model above corresponds to $\theta$ in (7) and $\beta_{/\theta}$ corresponds to $[a_0 \;\; a_1]$. The AXE approximation is as described in Section 2.2, where $\tilde{Y} = \log(E[\eta_j|Y])$, to account for the additional offset term which is not modeled by the GLMM.

## D.6 Scottish respiratory disease (SRD)

The Scottish respiratory disease data consists of annual observed respiratory-related hospital admissions in the $J = 271$ Intermediate Geographies (IG) of the Greater Glasgow and Clyde health board from 2007 - 2011; the yearly average modeled concentrations of particulate matter less than 10 microns ($\mathtt{PM}_{10}$); the average property price in hundreds of thousands of pounds (`Property`); the proportion of the working-age population who receive an unemployment benefit called the Job Seekers Allowance (`JSA`); the expected number of hospital admissions, $E_{tj}$, which is modeled as an offset-term; and the adjacency matrix $A$, where $A_{ii} = 0$, $A_{s_j i} = A_{ji} = 1$ if $j$ and $i$ are neighboring districts and 0 otherwise. It is available through the `CARBayesST` package in R.

We use the spatio-temporal auto-regressive model in Rushworth et al. (2014), where

observed hospital admissions for a year $t$ and IG $j$ are modeled with a Poisson density,

$$Y_{tj} = \text{Poisson}(\eta_{tj} E_{tj})$$
$$\log(\eta_{tj}) = x'_{tj}\boldsymbol{a} + \alpha_{tj},$$

where $x_{tj}$ is a vector containing $\texttt{PM}_{10}$, `Property`, and `JSA` values for that year $t$ and IG $j$; and $\boldsymbol{a}$ is the vector of fixed effects. Within each year, spatial dependence among the corresponding vector of random effects $\boldsymbol{\alpha}_t = (\alpha_{t1}, \ldots, \alpha_{tJ})'$ is modeled with covariance matrix $\sigma^2 Q(\rho_j, A)^{-1}$, where

$$Q(\rho_j, A)^{-1} = \rho_j(\text{diag}(A\mathbf{1}) - A) + (1 - \rho_j)I_{s_j}, \quad \rho_j \in [0, 1),$$

which induces spatial auto-correlation and is a special case of a CAR model. Temporal auto-correlation is introduced among the $\alpha_t$ by the conditional density of $\alpha_t | \alpha_{t-1}$:

$$\alpha_t | \alpha_{t-1} \sim N(\rho_T \alpha_{t-1}, \sigma^2 Q(\rho_j, A)^{-1}), j \in \{2, \ldots, T\}.$$

The joint density of $\boldsymbol{\alpha} = (\alpha'_1, \ldots, \alpha'_T)'$ is

$$\boldsymbol{\alpha} \sim N(0, \sigma^2[(I - \rho_T H)'\text{blockdiag}(Q(\rho_j, W))(I - \rho_T H)]^{-1})$$
$$H = \begin{bmatrix} \mathbf{0} & \mathbf{0} \\ \boldsymbol{I}_{J(T-1)} & \mathbf{0} \end{bmatrix},$$

where $\rho_T$ is the temporal dependence parameter, $\rho_j$ the spatial dependence parameter, $I_{J(T-1)} \in I\!R^{J(T-1) \times J(T-1)}$ is the identity matrix, and $\mathbf{0}$ are matrices of 0s with dimensions such that $H \in I\!R^{JT \times JT}$ accounts for the temporal auto-correlation. The model is fit using the `ST.CARar()` function in `CARBayesST` with the default priors $\boldsymbol{a} \sim N(0, 100, 000)$, $\sigma \sim IG(1, 0.001)$, $\rho_T \sim U(0, 1)$, $\rho_j \sim U(0, 1)$.

Cross-validation is conducted along the $J = 271$ IGs thus $n_j = 5$ for all CV folds $j = 1, \ldots, J$. Using the notation of (7), $\theta_j = \alpha_j \in I\!R^5$, $\theta = \alpha$, and $\beta_{/\theta}$ corresponds to a. The AXE procedure is as described in Section 2.2, where $\tilde{Y} = \log\big(E[\eta_{s_j}|Y]\big)$, to account for the additional offset term which is not modeled by the GLMM. To save computation time and avoid inverting $V_{-s_j} \in I\!R^{JT \times JT}$ for each cross-validation fold, we numerically solve for

$V$ given the full data and use the Sherman-Morrison matrix equation to obtain $V_{-s_j}$:

$$V_{-s_j} = (V^{-1} - X'_{s_j}\Phi_{s_j}^{-1}X_{s_j})^{-1}$$
$$= V + VX'_{s_j}(\Phi_{s_j} - X_{s_j}VX'_{s_j})^{-1}X_{s_j}V.$$

# E LRR percentage curves for IJ-C, NS-C

LRR percentage curves for IJ-C and NS-C are included in Figure 1, in comparison to AXE. Note that IJ-C is omitted from the SLC and SRD results; this is because all IJ methods assume independence between $Y_i|\Xi_i$, unless they are bounded and discrete. Results for NS-C in SRD include only 5 out of 271 CV folds; the remainder are excluded due to computation time. Computation time for the 5 CV folds under NS-C was 24.5 hours.

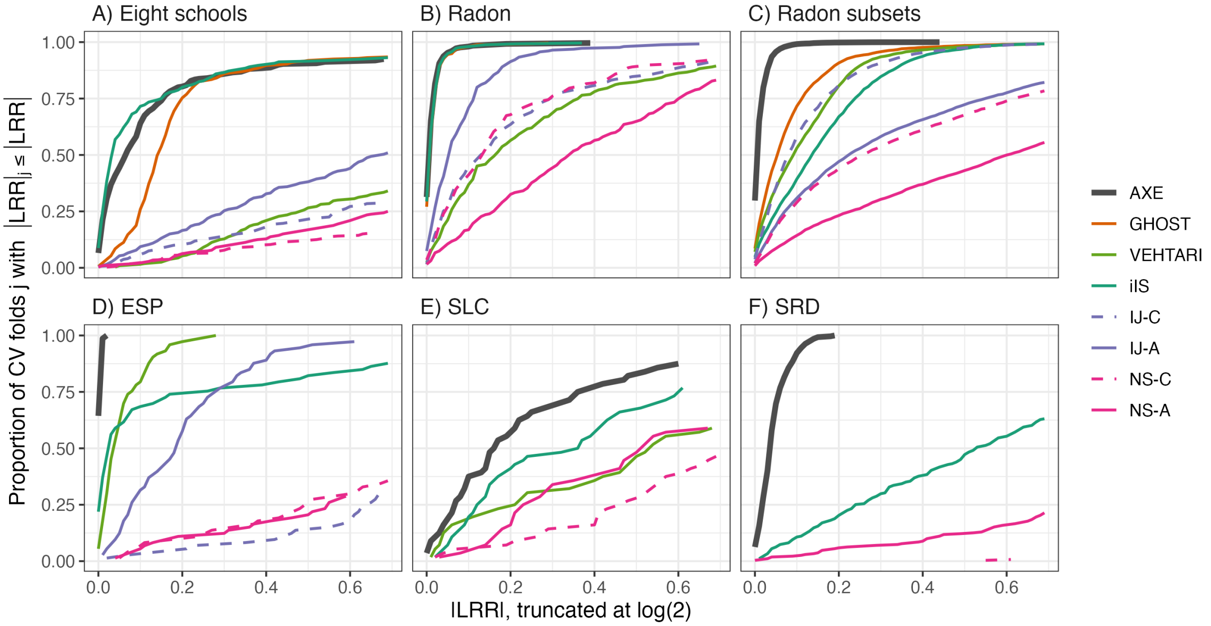


Figure 1: Line plots of the proportion of CV folds $j$ with $|\text{LRR}|_j \leq x$, for $x \in [0, \log(2)]$ on the x-axis. We refer to each line as an LRR percentage curve. Curves are colored based on the method used and are truncated at $\log(2) \approx 0.7$. LRR is calculated using the posterior mean for AXE and the posterior mode of $f(Y_{s_j}|\beta, \Sigma, \phi, Y_{-s_j})$ for IJ-C and NS-C.

# F Summary of LRR results

We record the mean and standard deviation of absolute LRRs for each example and method in Table 1 below.

Table 1: Mean and standard deviation (SD) of absolute log RMSE ratio (|LRR|, defined in (9)) for each leave-a-cluster-out CV approximation method and data set. LRRs are calculated for each CV loop. Vehtari was not applied to the SRD data due to the amount of time it would have taken.

| Data | AXE | | GHOST | | Vehtari | | iIS | | IJ-A | | NS-A | |
|---|---|---|---|---|---|---|---|---|---|---|---|---|
| | Mean | SD | Mean | SD | Mean | SD | Mean | SD | Mean | SD | Mean | SD |
| Eight schools | | | | | | | | | | | | |
| $\alpha < 2$ | 0.28 | 0.52 | 0.32 | 0.55 | 0.65 | 0.71 | 0.25 | 0.56 | 0.89 | 0.86 | 1.35 | 0.99 |
| $\alpha \geq 2$ | 0.12 | 0.23 | 0.19 | 0.20 | 2.31 | 1.03 | 0.11 | 0.19 | 1.02 | 0.98 | 1.85 | 1.29 |
| Radon | | | | | | | | | | | | |
| Model 1 | 0.04 | 0.24 | 0.05 | 0.27 | 0.16 | 0.25 | 0.05 | 0.24 | 0.11 | 0.27 | 0.41 | 0.36 |
| Model 2 | 0.02 | 0.05 | 0.02 | 0.04 | 0.32 | 0.29 | 0.02 | 0.04 | 0.08 | 0.13 | 0.56 | 0.51 |
| Model 3 | 0.01 | 0.01 | 0.02 | 0.01 | 0.32 | 0.30 | 0.01 | 0.01 | 0.10 | 0.10 | 0.34 | 0.33 |
| Radon subsets | | | | | | | | | | | | |
| Model 1 | 0.01 | 0.01 | 0.03 | 0.02 | 0.05 | 0.05 | 0.11 | 0.10 | 0.29 | 0.32 | 0.66 | 0.59 |
| Model 2 | 0.02 | 0.02 | 0.08 | 0.06 | 0.14 | 0.08 | 0.14 | 0.12 | 0.39 | 0.40 | 0.88 | 0.67 |
| Model 3 | 0.02 | 0.03 | 0.18 | 0.20 | 0.20 | 0.21 | 0.26 | 0.17 | 0.41 | 0.37 | 0.64 | 0.48 |
| ESP | 0.00 | 0.00 | ** | ** | 0.06 | 0.06 | 0.20 | 0.33 | 0.21 | 0.17 | 2.67 | 1.05 |
| SLC | 0.31 | 0.42 | ** | ** | 0.79 | 0.78 | 0.49 | 0.53 | ** | ** | 1.05 | 1.22 |
| SRD | 0.05 | 0.03 | ** | ** | * | * | 0.57 | 0.39 | ** | ** | 1.01 | 0.45 |

* Excluded due to computation time.

** Method does not apply.

# References


Bartholomew-Biggs, M., S. Brown, B. Christianson, and L. Dixon (2000). Automatic differentiation of algorithms. *Journal of Computational and Applied Mathematics 124*(1-2), 171–190.

Beirami, A., M. Razaviyayn, S. Shahrampour, and V. Tarokh (2017). On optimal generalizability in parametric learning. In I. Guyon, U. V. Luxburg, S. Bengio, H. Wallach, R. Fergus, S. Vishwanathan, and R. Garnett (Eds.), *Advances in Neural Information Processing Systems*, Volume 30. Curran Associates, Inc.

Gelfand, A. E. (1996). Model determination using sampling-based methods. *Markov Chain Monte Carlo in Practice*, 145–161.

Gelfand, A. E., D. K. Dey, and H. Chang (1992). Model determination using predictive distributions with implementation via sampling-based methods. Technical report, Stanford Univ. CA Dept. of Statistics.

Gelman, A. and J. Hill (2007). *Data Analysis Using Regression and Multilevel/Hierarchical Models*, Volume 1. Cambridge University Press New York, NY, USA.

Ghosh, S., W. T. Stephenson, T. D. Nguyen, S. K. Deshpande, and T. Broderick (2020). Approximate cross-validation for structured models. *arXiv preprint arXiv:2006.12669*.

Giordano, R., W. Stephenson, R. Liu, M. Jordan, and T. Broderick (2019). A swiss army infinitesimal jackknife. In *The 22nd International Conference on Artificial Intelligence and Statistics*, pp. 1139–1147. PMLR.

Goodrich, B., J. Gabry, I. Ali, and S. Brilleman (2018). rstanarm: Bayesian applied regression modeling via Stan. *R package version 2*(4), 1758.

Jaeckel, L. A. (1972). *The infinitesimal jackknife.* Bell Telephone Laboratories.

Kass, R. E. and D. Steffey (1989). Approximate Bayesian inference in conditionally independent hierarchical models (parametric empirical Bayes models). *Journal of the American Statistical Association 84*(407), 717–726.

Koh, P. W., K.-S. Ang, H. H. Teo, and P. Liang (2019). On the accuracy of influence functions for measuring group effects. In *Proceedings of the 33rd International Conference on Neural Information Processing Systems*, pp. 5254–5264.

Lee, D., A. Rushworth, and G. Napier (2018). Spatio-temporal areal unit modelling in r with conditional autoregressive priors using the carbayesst package. *Journal of Statistical Software 84*(9).

Li, L., S. Qiu, B. Zhang, and C. X. Feng (2016). Approximating cross-validatory predictive evaluation in Bayesian latent variable models with integrated IS and WAIC. *Statistics and Computing 26*(4), 881–897.

Maclaurin, D., D. Duvenaud, and R. P. Adams (2015). Autograd: Effortless gradients in numpy. In *ICML 2015 AutoML workshop*, Volume 238, pp. 5.

Marshall, E. and D. Spiegelhalter (2003). Approximate cross-validatory predictive checks in disease mapping models. *Statistics in Medicine 22*(10), 1649–1660.

Merkle, E. C., D. Furr, and S. Rabe-Hesketh (2019). Bayesian comparison of latent variable models: Conditional versus marginal likelihoods. *Psychometrika 84*(3), 802–829.

Owen, A. B. (2013). *Monte Carlo Theory, Methods and Examples.*

Rad, K. R. and A. Maleki (2020). A scalable estimate of the out-of-sample prediction error via approximate leave-one-out cross-validation. *Journal of the Royal Statistical Society: Series B (Statistical Methodology) 82*(4), 965–996.

Rubin, D. B. (1981). Estimation in parallel randomized experiments. *Journal of Educational Statistics 6*(4), 377–401.

Rushworth, A., D. Lee, and R. Mitchell (2014). A spatio-temporal model for estimating the long-term effects of air pollution on respiratory hospital admissions in greater london. *Spatial and Spatio-Temporal Epidemiology 10*, 29–38.

Stephenson, W. and T. Broderick (2020). Approximate cross-validation in high dimensions with guarantees. In *International Conference on Artificial Intelligence and Statistics*, pp. 2424–2434. PMLR.

Vanhatalo, J., J. Riihimäki, J. Hartikainen, P. Jylänki, V. Tolvanen, and A. Vehtari (2013). Gpstuff: Bayesian modeling with Gaussian processes. *Journal of Machine Learning Research 14*(Apr), 1175–1179.

Vehtari, A., A. Gelman, and J. Gabry (2016). Practical Bayesian model evaluation using leave-one-out cross-validation and WAIC. *Statistics and Computing 5*(27), 1413–1432.

Vehtari, A., T. Mononen, V. Tolvanen, T. Sivula, and O. Winther (2016). Bayesian leave-one-out cross-validation approximations for Gaussian latent variable models. *The Journal of Machine Learning Research 17*(1), 3581–3618.

Vehtari, A., D. Simpson, A. Gelman, Y. Yao, and J. Gabry (2015). Pareto smoothed importance sampling. *arXiv preprint arXiv:1507.02646*.

Wang, S., W. Zhou, H. Lu, A. Maleki, and V. Mirrokni (2018). Approximate leave-one-out for fast parameter tuning in high dimensions. In *International Conference on Machine Learning*, pp. 5228–5237. PMLR.
